# The Relationship between Height and Cognitive Function among Community-dwelling Elderly: Hallym Aging Study

**DOI:** 10.4178/epih/e2013002

**Published:** 2013-04-30

**Authors:** Shan Ai Quan, Jin-Young Jeong, Dong-Hyun Kim

**Affiliations:** 1 Department of Social and Preventive Medicine, Hallym University College of Medicine, Chuncheon, Korea.; 2 Hallym Research Institute of Clinical Epidemiology, Chuncheon, Korea.; 3 Korea Health Promotion Foundation, Seoul, Korea.

**Keywords:** Body height, Cognition, Hallym Aging Study

## Abstract

**OBJECTIVES:**

Height is known as an index that reflects the environment of the fetal, childhood, and adolescent periods, which affect adult health. This study was conducted to elucidate whether height is associated with cognitive impairment in community-dwelling elders in Korea.

**METHODS:**

The study subjects were recruited among community dwelling elderly individuals aged 65 or over who participated in the 2004 Hallym Aging Study. They were invited to a general hospital and were evaluated for socioeconomic status, smoking history, and various clinical measures. Cognitive function measurement was performed using the Korean-Mini Mental State Examination. Logistic regression was used to evaluate the association between height and cognitive function.

**RESULTS:**

After adjusting for potential covariates such as age and education, the smallest group was associated with higher risk of cognitive impairment compared with the tallest group among elderly men (odds ratio [OR], 4.20; 95% confidence interval [CI], 1.02-17.36), but not among elderly women (OR,1.65; 95% CI, 0.62-4.40).

**CONCLUSIONS:**

The reason for this difference according to sex may be explained by the differential effects of education on cognitive function by sex. A larger population-based prospective cohort study is needed to examine the association between height and cognitive function according to sex.

## INTRODUCTION

Height is representative of childhood health and nutrition, which affects the development of intelligence [1]. Childhood height is a strong predictor of cognitive function [2]. Likewise, if their health and nutritional status are good, adolescents are taller and have better cognitive abilities [3]. Fetal and early childhood nutritional status are also associated with the incidence of chronic disease in midlife [4]. Such disease can result in brain damage and thus in cognitive decline [5]. In one Finnish study of the school health examinations of males aged 6 and above, those who grew rapidly within a single year had a lower risk of coronary heart disease [4]. In the Nurses' Health Study [6], taller women had a lower risk of coronary heart disease. In some studies, greater height has been associated with reduced mortality [7,8], while in another study, short stature was associated with a lower rate of chronic disease and longer life expectancy [9]. In a study of elderly men (aged 76-95 years), it was suggested the onset of dementia could be postponed or prevented by maximizing growth of the body through improving childhood living conditions and adolescent nutrition [10], while in another study, taller elderly individuals had better cognitive functioning [1]. In the Health and Retirement Study, which was initiated to examine the physical and mental health, quality of life, and living environment in the population aged 50 and above since 1992, height was associated with various important health outcomes after 50 years of age in a follow-up study. In addition, taller elderly individuals had better cognitive functioning [1]. Likewise, height reflects childhood living conditions and is an important factor for healthy senescence.

There are only few domestic studies on the relationship between height and cognitive function. The study by Kim et al. [11] in women aged 60 and above is representative. One of the other studies is on the relationship between the lengths of extremities and dementia in an elderly population aged 65 and above [12], while another one is investigated the association between the length of the upper limbs and cognitive function [13].

Therefore, given the paucity of research in this area, this study investigated the relationship between height and cognitive function in a senior population residing in Chuncheon.

## MATERIALS AND METHODS

### Subjects

**Figure 1:**
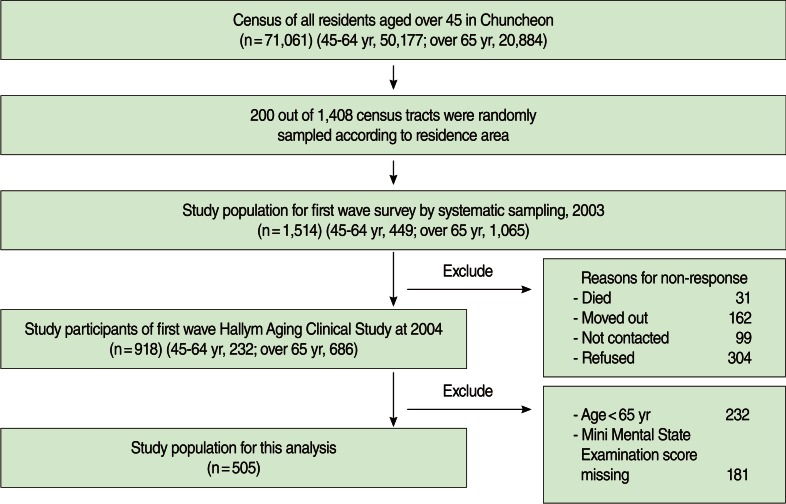
Flow chart of Hallym Aging Study.

The Hallym Aging Study was a cross-sectional study on the quality of life in a Korean elderly population in 2003. After randomly selecting 200 enumeration districts out of 1,408 based on the census of the population of Chuncheon City in 2000 and allocating a number of individuals to the sample based on the ratio of those aged 45 and above in each town, subjects were chosen by systematic sampling of the selected enumeration districts. In consideration of the production of stable epidemiological indicators and the effect of a long-term follow-up study in elderly population, with a proportion of 30% of the sample was assigned to those aged 45 to 64 and 70% to those aged 65 and above. The final number of survey participants was 1,514 (449 people aged 45-64 and 1,065 people aged 65 and above), and they were subjects of the first clinical in-depth survey performed in 2004. Nine hundred eighteen people responded to the survey while 596 people did not due to death, refusal, inability to contact, or relocation. In the end, 505 people were included for the analysis after excluding 232 people below 65 years of age and 181 people missing cognitive functioning test results (Figure 1). Details of the selection method are described in a previously published article [14].

### Data collection and measurement

#### Korean-Mini Mental State Examination

The Korean version of the Mini Mental State Examination (K-MMSE) developed by Kang et al. [15] has a total of 23 items with 30 possible points including time-orientation with 5 points, place-orientation with 5 points, memory registration with 3 points, concentration and calculation with 5 points, reminiscence with 3 points, language with 8 points, and visual organization with 1 point. In a validation study of hospitalized dementia patients, the sensitivity of the diagnosis for dementia was 70.3 to 82.7% and its specificity was 91.3% by applying a cut-off value of 23/24. In this study, the K-MMSE was administered by trained interviewers in a separate place where the participant would not be disturbed for about 10 minutes. Based on the K-MMSE scores, the two groups were divided into a cognitive impairment group with scores of less than 23 points and a normal group with scores of 24 and above.

#### Height measurement

Subjects were barefoot or wore light socks, and then they wore light clothes to enable the correct posture. They stood up straight with the chest out on a horizontal surface, and their heels, hips, back, and the back of their head against a vertical line. They faced forward. Both arms allowed to hang naturally without force, with the palms facing the thighs. The heels were positioned close together, and the inside angle of the feet was about 60 degrees. After the knees were positioned close together without overlapping and the participant took a deep breath, a horizontal plate was lowered to sit on top of the head. Heights were measured to the nearest 0.1 cm.

#### Socio-economic status

Demographic characteristics including sex, age, and educational level were collected by survey. For the education level, the extent of education was measured as a continuous variable. Individuals were then classified into "no formal education," "1 to 5 years of education," and "6 years of education or more."

#### Lifestyle

The subjects' smoking and drinking histories were measured as behavioral variables. For the smoking history, individuals were classified into "non-smoking," "current smoking," and "past smoking" groups. To examine the drinking history, the categories were "non-drinking," "current drinking," and "past drinking."

### Statistical analysis

The difference in the distribution between the normal group and the cognitive impairment group by general characteristics was analyzed using a chi-squared test. The Wilcoxon test was used to analyze the differences in average body measurements and cognitive functioning test results. A multivariate logistic regression analysis was performed by dividing height into quartile ranges to analyze the relationship between cognitive impairment and height. Considering differences by sex, analyses were performed separately for the males and the females. SAS version 9.1 (SAS Inc., Cary, NC, USA) was used for the statistical analysis; the significance level was 0.05.

## RESULTS

In the males, more educated subjects tended to be in the normal group compared to uneducated subjects. Uneducated subjects of the female group were more distributed in the cognitive impairment group, and educated subjects were in the normal group. The cognitive impairment group in males was much more likely to contain subjects with a current smoking status. The average height in the males was 159.4 cm in the cognitive impairment group and 162.9 cm in the normal group. For the females, the average height was 146.8 cm in the cognitive impairment group and 149.4 cm in the normal group. These results were statistically significant (p<0.001). Height was divided into quartile ranges (Q1-Q4) by sex. In the males, the shortest group was highly distributed, with 46.5% in the cognitive impairment group, followed in sequence by Q2 (=Q3) and Q4. In the cognitively normal group, individuals were evently distributed by height. In the females, as in the males, there were an increasing number of subjects in the cognitive impairment group as height decreased. The proportion of normal subject in the shortest group, on the other hand, was 17.7%, while that of normal subjects in the tallest group was 29.2%. This meant that taller subjects tended to be in the normal group (Table 1).

**Figure 2:**
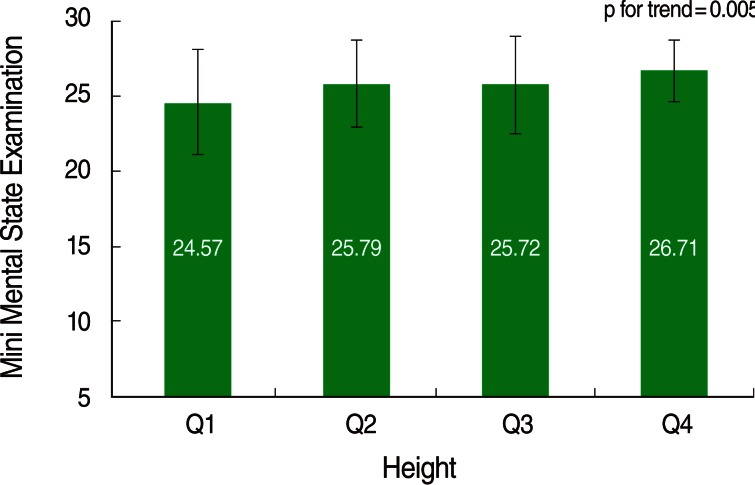
Mini Mental State Examination score according to height quartile group among men.

**Figure 3:**
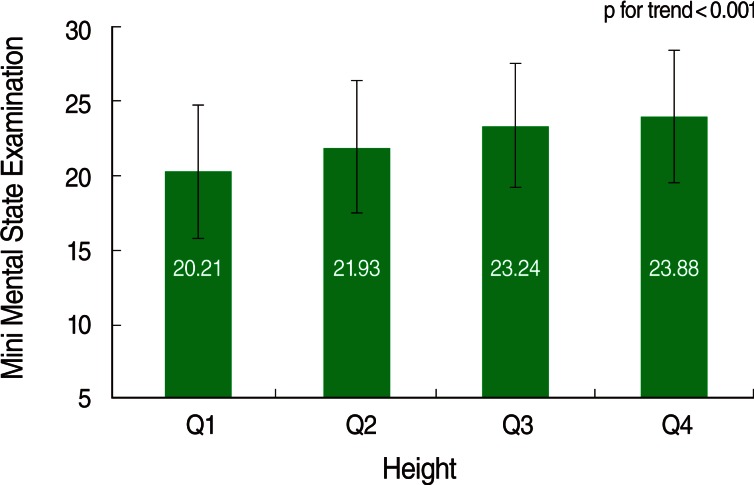
Mini Mental State Examination score according to height quartile group among women.

Cognitive function scores in each height quartile range were divided by sex. In both sexes, greater height was associated with higher cognitive scores, and this was statistically significant under the significance level of 0.05 (Figures 2 and 3).

A logistic regression analysis was performed to investigate the relationship between cognitive function and height. After correction for age, education, and smoking factors, the risk of cognitive impairment was 4.20 times higher in the shortest group (Q1) than in the tallest group (Q4) of the males (95% confidence interval [CI]=1.02-17.36) (Table 2). In the females, the risk of cognitive impairment was 4.29 times higher in the shortest group than in the tallest group (95% CI=2.07-8.92); however, there was no relationship after correction for age and education factors (OR, 1.65; 95% CI, 0.62-4.40) (Table 2). Because short height and cognitive function may be affected by medical history, the data was analyzed with stratification by diseases; however, there was no significant difference in the results before and after stratification (data not shown).

## DISCUSSION

In this study, a relationship between height and cognitive impairment was shown in the males with correction for age and educational factors; but there was no relationship in the females. In various recent studies, height has been shown to be an indicator of childhood living environment, and childhood health condition strongly affects health in adulthood and senescence [16].

Reviewing previous studies on the relationship between height and cognitive function, it was reported that height was associated with Alzheimer's disease and vascular dementia in a cohort study of Israeli males [10]. Another British birth cohort study [17] has suggested that height in all ages showed a positive relationship with cognitive function in 3,000 people. In a study of Japanese males who moved to the US, short height in early life indicated a stronger potential for cognitive impairment [18]. In addition, it was reported that height had a positive association with cognitive impairment in Korean females [11]. In most studies, it has been suggested that height has a relationship with cognitive function; however, no relationship was shown for the females in this study. Mak et al. [19] suggested that the length of the lower extremities had no association with cognitive function in 290 males and females aged 55-75 grouped by three-year intervals in a follow-up study. Furthermore, in the study of Jeong et al. [13] on 235 males and females aged 65 and above residing in Namwon City, South Korea, although there was no association with cognitive impairment, the length of the arms showed a relationship with cognitive impairment; thus, it was suggested that the length of the arms was a more stable predictor of cognitive function.

Childhood living environment and cognitive development level affect educational attainment, school achievement, and eventual choice of occupation [1]. Some part of the reason that the childhood living environment affected cognitive function in senescence may be that children with high socioeconomic status had a high education level [1]. The males in the generation that is currently elderly had a greater opportunity to be educated than the females of the same cohort. The difference by sex could be explained by the complex effects of education and social roles rather than by sex itself [20]. With a life course perspective, women have fewer chances to obtain social positions, opportunities, and resources during their whole life course, and this result accumulated [21]. Likewise, the difference in cognitive function by sex is caused by the different opportunities for education, and thus there was no relationship between height and cognitive function with the correction for educational factors in the females.

The genetic factor contributes only 20% to height, and the effect of the genetic factor is decreased by poor environment [22]. Poor childhood health and nutrition affect physical health in adulthood, and thus cognitive function. Furthermore, these conditions cause diabetes mellitus, hypertension, and cardiovascular diseases, which are related to cognitive impairment of the elderly [23]. At the same time, a cohort study of twins suggested that height had a strong association with a genetic factor as well as childhood environmental factors [24]. Moreover, in a Danish cohort study [25], it was reported that the relationship between height and cognitive function decreased in developed social environments after the Second World War. As Korean socioeconomic conditions have continued to develop rapidly, ongoing studies on the relationship between height and cognitive function are needed.

It is difficult to explain the direction of cause and effect between height and cognitive function with this study because it is a cross-sectional study. Because cognitive function is affected by various factors and changes over time, it can be concluded that a cohort study to investigate cognitive function and the factors affecting it would be more effective than a cross-sectional study.

This study was performed to examine the relationship between height and cognitive function in the elderly population aged 65 and above residing in Chuncheon City, South Korea. In the males and the females, taller elderly individuals had a better average cognitive functioning score. With the logistic regression analysis, the risk of cognitive impairment in the shortest group was higher than in the tallest group for the male, while, no relationship was shown in the female.

## Figures and Tables

**Table 1:**
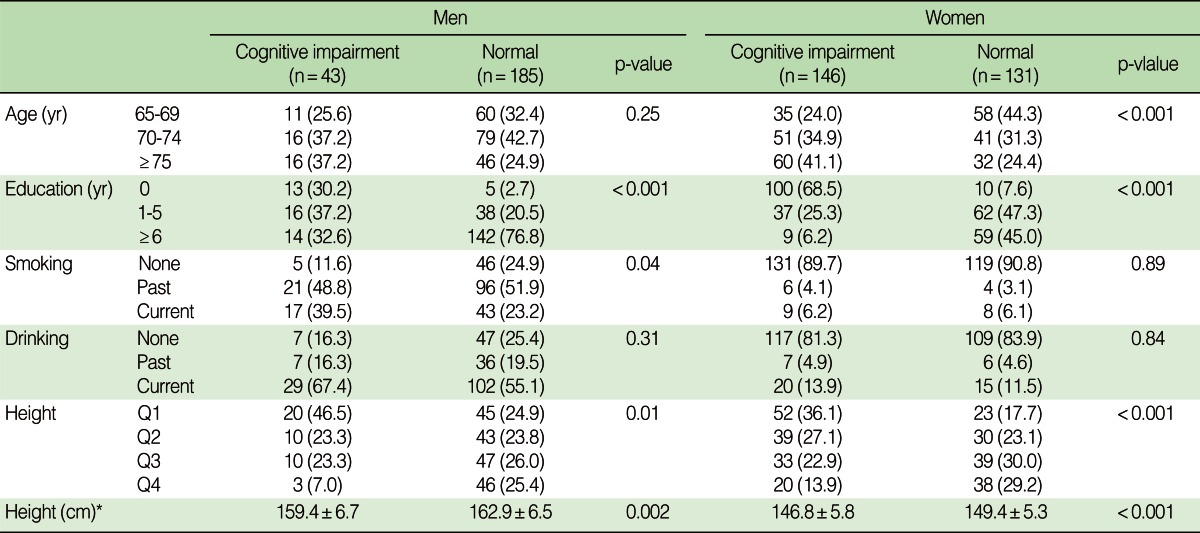
Characteristics of participants by cognitive function

Values are presented asnumber (%) or mean±SD. ^*^Wilcoxon's rank sum test.

**Table 2:**
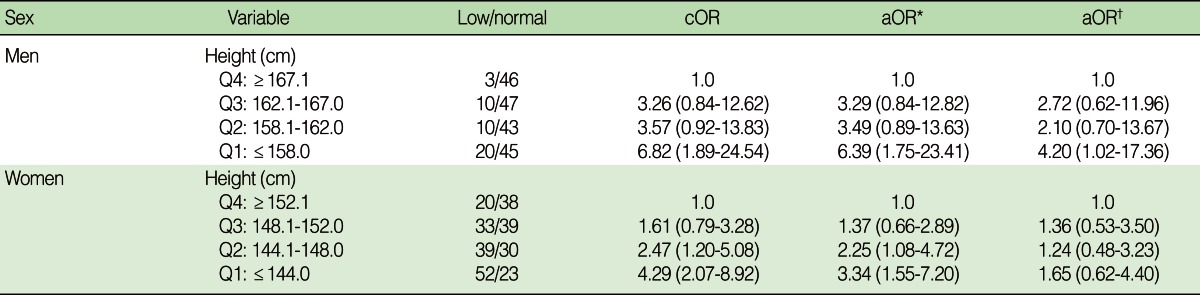
Effect of height on decreased cognitive function according to sex

Values are presented as number or odds ratio (confidence interval). Odds ratio (95% confidence interval). cOR, crude odds ratio; aOR, adjusted odds ratio. ^*^Adjusted for age; ^†^Adjusted for age, education, and smoking (only for men).
